# Immediate versus deferred antiretroviral therapy in HIV-infected patients presenting with acute AIDS-defining events (toxoplasmosis, *Pneumocystis jirovecii*-pneumonia): a prospective, randomized, open-label multicenter study (IDEAL-study)

**DOI:** 10.1186/s12981-019-0250-2

**Published:** 2019-11-15

**Authors:** Guido Schäfer, Christian Hoffmann, Keikawus Arasteh, Dirk Schürmann, Christoph Stephan, Björn Jensen, Matthias Stoll, Johannes R. Bogner, Gerd Faetkenheuer, Jürgen Rockstroh, Hartwig Klinker, Georg Härter, Albrecht Stöhr, Olaf Degen, Eric Freiwald, Anja Hüfner, Sabine Jordan, Julian Schulze zur Wiesch, Marylyn Addo, Ansgar W. Lohse, Jan van Lunzen, Stefan Schmiedel, the IDEAL study group

**Affiliations:** 1 Infectious Diseases Clinic, University Medical Center Hamburg-Eppendorf, Hamburg, Germany; 2 1st Medical Department, Section Infectious Diseases & Tropical Medicine, University Medical Center Hamburg-Eppendorf, Hamburg, Germany; 3 ICH Study Center GmbH & Co KG, Hamburg, Germany; 4 Department for Infectious Diseases, Vivantes Auguste-Viktoria-Klinikum, Berlin, Germany; 5 Department for Pneumology and Infectious Diseases, Charité Universitätsmedizin, Berlin, Germany; 6 2nd Medical Department, Section Infectious Diseases, University Medical Center, Frankfurt am Main, Germany; 7 Department for Gastroenterology, Hepatology, Infectious Diseases, Universitätsklinikum Düsseldorf, Düsseldorf, Germany; 8 Department for Immunology and Rheumatology, Medizinische Hochschule Hannover, Hannover, Germany; 9 Department for Infectious Diseases, Mediznische Klinik und Poliklinik IV der Universität München, Munich, Germany; 10 1st Medical Department, Section Infectious Diseases, Universitätsklinikum Köln, Cologne, Germany; 11 Medical Department, Section Infectious Diseases, Universitätsklinikum Bonn, Bonn, Germany; 12 Department for Infectious Diseases, Julius Maximilians University, Würzburg, Germany; 13 Department for Infectious Diseases, University Hospital, Ulm, Germany; 14 ifi-Institute for Interdisciplinary Medicine, Hamburg, Germany; 15 Institute for Medical Biometry and Epidemiology, University Medical Center Hamburg-Eppendorf, Hamburg, Germany; 16 ViiV Healthcare, Research Triangle, USA

**Keywords:** Late presentation, Opportunistic infections, *Pneumocystis jirovecii*, PCP, Cerebral toxoplasmosis HIV, Diagnosis, Treatment

## Abstract

**Background:**

To evaluate clinical outcomes after either immediate or deferred initiation of antiretroviral therapy in HIV-1-infected patients, presenting late with pneumocystis pneumonia (PCP) or toxoplasma encephalitis (TE).

**Methods:**

Phase IV, multicenter, prospective, randomized open-label clinical trial. Patients were randomized into an immediate therapy arm (starting antiretroviral therapy (ART) within 7 days after initiation of OI treatment) versus a deferred arm (starting ART after completing the OI-therapy). All patients were followed for 24 weeks. The rates of clinical progression (death, new or relapsing opportunistic infections (OI) and other grade 4 clinical endpoints) were compared, using a combined primary endpoint. Secondary endpoints were hospitalization rates after completion of OI treatment, incidence of immune reconstitution inflammatory syndrome (IRIS), virologic and immunological outcome, adherence to proteinase-inhibitor based antiretroviral therapy (ART) protocol and quality of life.

**Results:**

61 patients (11 patients suffering TE, 50 with PCP) were enrolled. No differences between the two therapy groups in all examined primary and secondary endpoints could be identified: immunological and virologic outcome was similar in both groups, there was no significant difference in the incidence of IRIS (11 and 10 cases), furthermore 9 events (combined endpoint of death, new/relapsing OI and grade 4 events) occurred in each group.

**Conclusions:**

In summary, this study supports the notion that immediate initiation of ART with a ritonavir-boosted proteinase-inhibitor and two nucleoside reverse transcriptase inhibitors is safe and has no negative effects on incidence of disease progression or IRIS, nor on immunological and virologic outcomes or on quality of life.

## Introduction

Among the approximately 36.7 million people infected with the human immunodeficiency virus (HIV) worldwide [1], the population of patients presenting late, at advanced disease stages, is still a significant and challenging medical issue. Current evidence points to high rates of late presenters across Europe—between 15 and 63.6% of all new HIV cases [2–10]. Moreover, the proportion of late presenters is increasing or at best stagnant in many countries, despite several attempts to encourage earlier diagnosis [5, 11–15].

Late diagnosis of HIV infection often only follows after patients present with opportunistic infections (OI) requiring immediate treatment, a situation which poses numerous management dilemmas and risks due to overlapping drug toxicities, potential pharmacokinetic interactions with antiretroviral drugs and the risk of developing immune reconstitution inflammatory syndrome (IRIS) [14, 16–21]. There has been much debate about the optimal timing of antiretroviral therapy (ART) initiation in ART naïve patients presenting with an AIDS-defining event although clinical trials are lacking. Some recent studies suggest that a delay of initiation may be associated with poorer outcomes and more rapid HIV disease progression [14, 19, 22–27]. However, there is conflicting evidence in favor for a deferred initiation of ART in patients diagnosed with cerebral tuberculosis and cryptococcal meningitis [28, 29].

Late presenting patients with OIs are underrepresented or excluded in most ART therapy-studies and a limited number of studies address the optimal timing of ART initiation during the course of treatment of an OI. An active AIDS-defining illness is almost always among the exclusion criteria. This situation has resulted in a striking lack of evidence concerning the antiretroviral treatment of such patients. Consequently, the best antiretroviral regimen and the best timing for starting antiretroviral therapy in treatment-naive patients with advanced infection have not yet been established [14] and guidelines for specific antiretroviral treatment for late-presenting patients are lacking [22].

The objective of the current study was to compare the rates of clinical progression in early versus deferred initiation of ART in two study groups with treatment naïve patients, presenting with pneumocystis pneumonia (PCP) and toxoplasmic encephalitis (TE). The ART consisted of atazanavir/ritonavir (ATV/r) and tenofovir-disoproxil fumarate (TDF)/emtricitabine (FTC).

## Methods

### Trial design

This study (NCT 01417949, clinicaltrials.gov/ct2/show/NCT01417949, registered August 16th 2011) was planned and conducted as a phase IV, multicenter, prospective, randomized open-label clinical trial. A biostatistician was involved in planning of the study. Recruitment was conducted in 16 specialized infectious diseases care clinics in Germany, recruitment period was September 2011 until May 2015. All feasible patients in these centers were screened for inclusion.

### Subjects

All patients gave written informed consent. Inclusion criteria were defined as HIV-1-infected adults and either ART-naïve or patients with no antiretroviral therapy for at least 6 months prior to screening and no evidence for prior virologic failure due to resistance against nucleoside reverse transcriptase inhibitors (NRTIs) and protease inhibitors (PIs), who have developed either pneumocystis pneumonia (PCP) or toxoplasma encephalitis (TE). Exclusion criteria included renal failure (defined as CrCl < 60 mL/min), patients who were unable to initiate ART or with current contraindications against ATV/r, AIDS-defining events other than PCP or TE, pregnancy or women of childbearing potential wanting to become pregnant. After statistical calculations we planned 210 patients to include into the study to gain statistically significant evidence. The sample size calculation was carried out with Pass 2008 (Power Analysis and Sample Size Software; NCSS, LLC. Kaysville, Utah, USA). The underlying assumption was a reduction of the primary event rate from 25 to 10% with a statistical power of 80% and a type I error of 5% within a balanced study design. In addition, a maximum dropout rate of 7.5% is assumed for the 194 necessary patients. This results in the final total number of 210 subjects.

### Study centers

All patients were recruited in an in-hospital setting. All study centers were HIV-care experienced infectious-diseases units of hospitals with associated outpatient care centers in urban settings.

### Study medication and schedule

Antiretroviral treatment consisted of Ritonavir boosted Atazanavir (ATV/r) plus 2 nucleosidic non reverse transcriptase inhibitors (NRTIs). The immediate treatment group (IT) started ATV/r + 2 NRTIs immediately after HIV/AIDS diagnosis (as soon as possible but no later than 7 days after initiation of OI treatment). The deferred treatment arm (DT) started ART after completion of OI treatment, which was achieved for PCP at the earliest at day 21, for TE at the earliest at day 28, but no later than 6 weeks after randomization. Patients were followed up for a total of 24 weeks. Bristol-Myers Squibb provided the study sites with Norvir^®^ and Reyataz^®^ for this clinical trial. Randomization was performed by the principal investigator within the electronic case report form (eCRF). After entering the information that the patient had given written informed consent the decision when to start ART (“immediate” or “deferred”) was provided by the e-CRF system. The day of randomization was considered to be baseline in both arms. Follow up period was over 24 weeks (with visits every 4 weeks). The in-patient care not directly related to the study procedures was up to the specific study site (for example the choice of treatment of the OI or the time of discharging the patients). In other words, the decision to discharge a patient was made on clinical grounds. There was a documentation of the hospitalization days (see next section).

### Primary and secondary endpoints

The primary endpoint was defined as clinical progression [death, all new or relapsing OI, grade 4 (G4) clinical endpoint] within 24 weeks after inclusion in the study. For G4 events, standardized toxicity grading tables were used (https://rsc.niaid.nih.gov/sites/default/files/table-for-grading-severity-of-adult-pediatric-adverse-events.pdf). For abnormalities not found in the toxicity tables, a grade 4 event was defined as potentially life-threatening (extreme limitation in activity, significant assistance required; significant medical intervention/therapy required, hospitalization or hospice care probable). Patients who dropped out of study observation before the end of week 12 were counted as clinical progression (intention-to-treat analysis). Secondary endpoints were defined as incidence of immune reconstitution inflammatory syndrome (IRIS) within 24 weeks after inclusion, virological outcome (proportion of patients achieving a viral load below 400 or 50 copies/mL at 24 weeks, changes of the antiretroviral regimen (for lack of efficacy or toxicity), parameters for quality of life (SF 36 score at inclusion and at 24 weeks) and adherence of ART (pill count) and the number of hospitalization days.

### Statistical considerations

Originally intended statistical method (log-likelihood Chi-square test for primary, Wilcoxon t- and Chi-square test for secondary endpoints) could not be applied every time due to low inclusion of patients (see results). In those cases, Chi-square was replaced by Fisher’s exact test. All analyses were performed using SAS 9.4 (SAS Institute 2016, Cary, NC). All tests were fully functional despite the small study populations.

### Ethics statement

All participating sites had local ethical approval due to national laws. All subjects provided written informed consent.

## Results

**Fig. 1:**
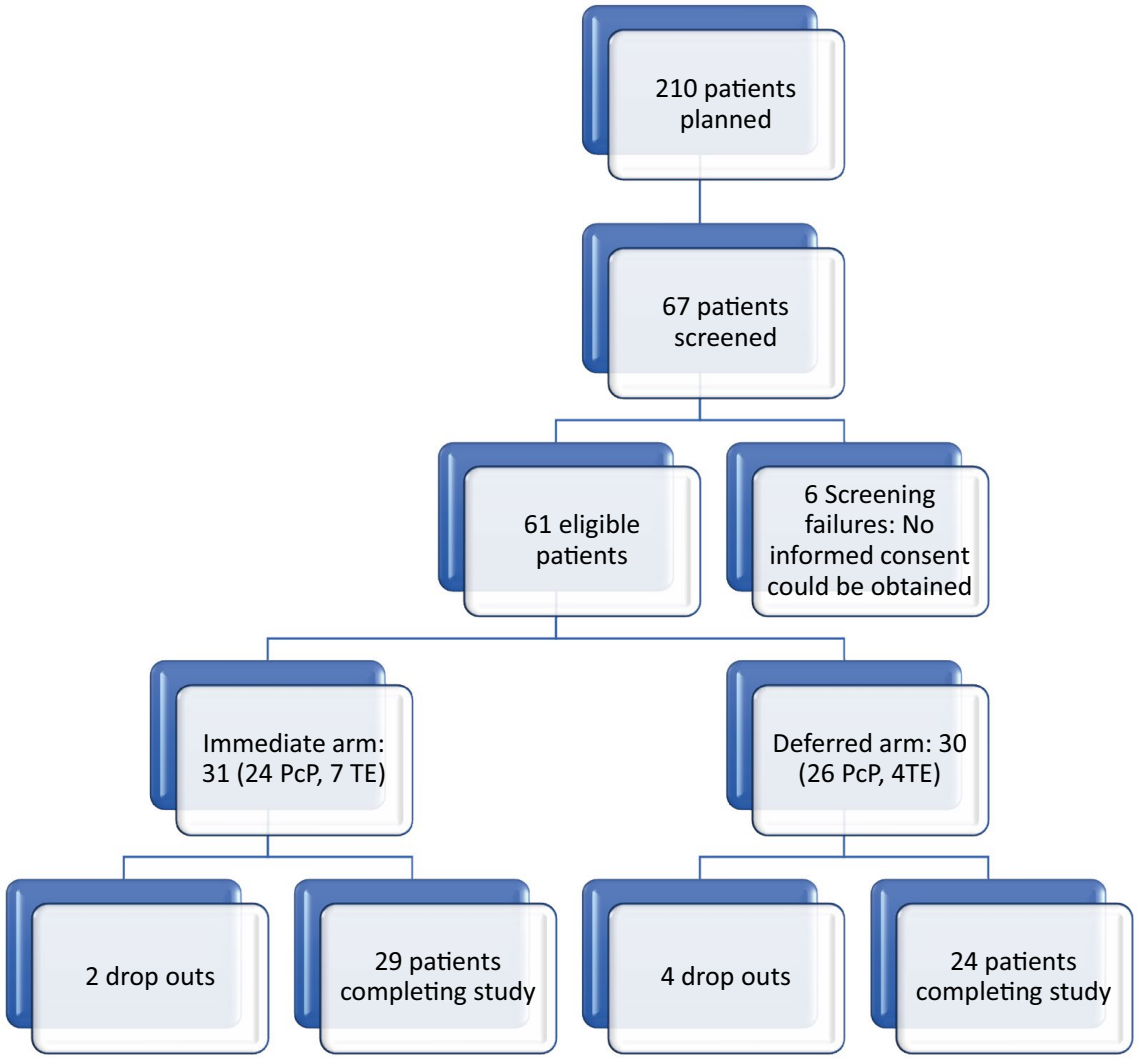
Study subject enrollment and discontinuations

We screened 67 patients overall, 6 patients couldn’t be included (either no informed consent could be obtained due to psychiatric impairment or due to comorbidities and comedication, 4× TE, 2× PCP with severe disease/sepsis). 31 patients were randomized to the immediate arm and 30 patients to the deferred arm, of whom 29/31 and 24/30 completed the follow up period of 24 weeks. Mean age, gender- and race-distribution were comparable in both groups (see Tables 1 and 2 and Fig. 1). Body mass index, blood pressure, pulse- and respiratory rate at baseline and at last follow up (not shown in the tables) showed no significant differences. 11 patients with toxoplasmosis and 50 patients with PCP were included. There were no significant differences in adherence to treatment or treatment interruptions (< 10% of patients had interruptions in treatment). All patients received ATV/r + TDF/FTC (no other NRTI-backbone was chosen in the included study population). In May 2015, the study was terminated due to low recruitment (decision was made during a prolongation period by the principal investigator and the sponsor together).

**Table 1:** Demographics, clinical baseline characteristics of the study populations

Parameter	Sub-parameter	Immediate treatment	Deferred treatment	p-value (if applicable)
Age (mean ± SD) (years)		42.9 (± 8.6)	42 (± 10.4)	0.714
Gender-no./total-no. (%)	Male	29/31 (93.5)	27/30 (90.0)	0.671
	Female	2/31 (6.5)	3/30 (10.0)	
Race-no./total no. (%)	White	28/31 (90.3)	26/30 (86.7)	0.833
	Black-African	3/31 (9.7)	4/30 (13.3)	
Viral load before randomization	Mean (cop/mL)	447,579	570,020	
CD4 cells before randomization (baseline)	Mean (absolute)	27	42	
PCP (n = number of patients)		24	26	
TE (n = number of patients)		7	4

**Table 2:** Demographics, clinical characteristics and outcome (week 24) of the study populations

Parameter	Sub-parameter	Immediate treatment	Deferred treatment	p-value (if applicable)
Event^a^ (frequency row %)	No event	22 (70.97)	21 (70.00)	0.934 (computed by Chi-square)
	Event	9 (29.03)	9 (30.00)	
Hospitalization (days)	Mean (absolute)	33.2 ±* 19.1*	34.57 ± 23.6	0.6125 (computed by t-test)
	Range (absolute)	141	205	
Incidence of IRIS (%)	Yes	11 (35.48)	10 (33.33)	0.8597 (computed by Chi-square)
	No	20 (64.52)	20 (66.67)	
Virological outcome week 24 (more than 400 or 50 copies/mL)	Number of patients with HIV RNA > 50 cop/mL	12	12	0.6740 (computed by Chi-square)
	With HIV RNA > 400 cop/mL	1	1	1.0 (computed by Fisher’s Exact test)
Immunological outcome (week 24): CD4 cells	Absolute mean	126 (±* 42.1*)	137 (±* 44.0*)	0.6421 (computed by t-test)
	Delta mean	99	95	
Quality of life	SF 36 body score (sum of scores)	5706	5772	0.7978
	SF 36 psychological score (sum of scores)	5622	5106	0.7978 (each computed by Wilcoxon test)
AE-severity by treatment (%)	Grade 1 event	61 (22.26)	73 (26.64)	0.4682 (computed by Fisher’s Exact test)
	Grade 2 event	53 (19.34)	55 (20.07)	
	Grade 3 event	6 (2.19)	11 (4.01)	
	Grade 4 event	3 (1.09)	6 (2.19)	
	Unknown severity	1 (0.38)	5 (1.82)	
	Total	124 (45.26)	150 (54.74)	
PCP		4 events	6 events	0.6372 (number of events between TE/PCP; computed by Chi-square)
TE		5 events	3 events	

Italicized values are standard-deviations of the mean-values

^a^Definition of event: death, all new or relapsing OI, other grade 4 (G4) clinical endpoint within 24 weeks (see “Methods” section)

### Clinical progression (primary endpoint)

No significant differences in clinical progression between groups were detected: 9 of 31 patients in the immediate and 9 of 30 patients in the deferred group (p-value: 0.9340) had all new or relapsing OI or grade 4 events diagnosed. A new or relapsing opportunistic infection occurred in 4 of 31 patients in the immediate arm and in 1 of 30 patients in the deferred arm. In total, seven (7) grade 4 adverse events occurred throughout the observation period: in 3 of 31 immediate group patients and in 4 of 30 deferred group patients. Serious adverse events were reported for either 2 out of 31 immediate group patients and 6 out of 30 deferred group patients dropped-out.

Of the 61 randomized patients, 8 of 11 patients with TE and 10 of 50 patients PCP had an event, however, these were similarly distributed between the IT and DT groups (see Table 2).

### Adverse events

Overall, the patients experienced 274 adverse events (AE). 242 (88%) were graded as grade 1 or 2 adverse events. Most prevalent AE were: allergy to antibiotics (12), herpes infection (8× herpes simples, 3× herpes zoster), mucosal candida infection (8), nausea (7), fever (7), respiratory tract infections (bronchitis, pneumonia) (5), cytomegalovirus (CMV) infection (5), diarrhea (5), Kaposi sarcoma (3).

The distribution of grade 1 and 2 adverse events was relatively equal between the study arms. Although not statistically significant, grade 3 and 4 events appeared to be more common in the DT group (see Table 2).

24 AE (8.76%) were assessed as related to study treatment (18 grade 1, 5 grade 2 and 1 grade 4). The dose of study drug was not changed for grade 1 and 2 AEs however, drug treatment was discontinued after the grade 4 AE (the patient was in the DT group). No serious adverse drug reaction (SADR) or suspected unexpected serious adverse reaction (SUSAR) was recorded in either treatment group. The drop-out rate after treatment initiation was 2/31 (6.45%) in the IT group and 6/30 (20%) in the DT group.

In total, 18 SAEs occurred during the clinical trial. Two of them, occurring within the same patient, were reported as fatal (a 35-year-old man in the deferred treatment group, was diagnosed with a T-cell lymphoma and then later died due to septic multiorgan failure).

### Secondary endpoints

No significant difference or trends were detectable amongst secondary endpoints. This includes hospitalization days, IRIS incidence, immunological and virologic outcome, the proportion of patients with changes in the antiretroviral regimen or the SF36-scores (Table 2).

## Discussion

This trial was conducted to gain more evidence regarding the ideal timing of initiation of ART in late presenting HIV positive patients who present with either PCP oder TE.

The major strength of this study is that it provides data for the important question when to start ART in a very vulnerable group of HIV-infected patients with two common opportunistic infections.

The protease-inhibitor (PI) atazanavir/ritonavir (ATV/r) was chosen as core agent, recommended in several both national and international guidelines at the time of study planning and initiation 2010–2011 (WHO, EACS, CDC) [1, 30, 31]. ATV/r was chosen as a once daily (at this time) state of the art regimen, as it has a higher genetic barrier compared to e.g. efavirenz and causes a rapid decline in viral load [32]. Retrospectively seen, this choice probably contributed to the low recruitment, as possible drug–drug interactions.

In general, the study medication was well tolerated, most adverse events were mild. There were no unsuspected adverse events related to the study medication. Only few adverse events were severe adverse events and only few drop-outs occurred. The total number of these events (drop-out, AE grade 4, SAE) were equally low in both treatment groups, considering the severity of the underlying condition.

A review of the available literature regarding timing of ART in HIV positive patients with opportunistic infections was performed. To our knowledge only one randomized clinical trial, performed by the AIDS Clinical Trial Group (ACTG 5164) seems similar and comparable to our study population and setting [22]. In this trial a total of 282 subjects (39 sites within the United states and Johannesburg, South Africa) were randomized to initiate ART immediately or after OI treatment. The study provided lopinavir/ritonavir [LPV/r], emtricitabine [FTC], tenofovir disoproxil fumarate [TDF], and stavudine [d4T]. However, any antiretroviral agent approved by the FDA for the initial treatment of HIV was allowed. A boosted PI with two NRTIs was used in 89% of subjects in the immediate arm and 85% of subjects in the deferred arm while NNRTI-based regimens with two nRTIs were used in 11% and 16%, respectively.

There was no statistically significant difference in the primary endpoint and both arms achieved similar CD4 T-cell counts and viral load declines by week 24. Importantly, the immediate arm had fewer deaths/AIDS progressions (p = 0.035), longer time to death/AIDS progression (stratified HR = 0.53, p = 0.02), and shorter time to achieving an increase in CD4 T-cell counts to > 100/μL (11.8 versus 4.2 weeks). The authors concluded that, although there was no significant difference between immediate and deferred ART in the primary endpoint (clinical and virologic response), immediate ART reduces death/AIDS progression over 48 weeks. However, there are major differences in the study settings to our trial because different antiretroviral regimens have been used and heterogeneous opportunistic infections were allowed. The initiation of ART in the immediate arm was 12 days, compared to 45 days in the deferred arm. To get better comparability, we’ve chosen a defined ART regimen with boosted Atazanavir as the third agent. This was an appropriate, frequently recommended and used regimen at time of study planning 2010–2011 [30, 33]. We focused on the two most frequently occurring OI in high income countries, which is another strength of this study.

This study shows no evidence that an immediate antiretroviral combination treatment with ATV/r and 2 NRTIs (TDF/FTC) in patients with an acute AIDS-defining opportunistic infection (toxoplasmosis or pneumocystis pneumonia) has any positive or negative effect on the overall outcome of patients with advanced HIV-related immunosuppression (CDC class C). The primary endpoint ‘clinical progression’ (including death, all new or relapsing OI, and other G4 clinical endpoints within 24 weeks) occurred equally in both treatment arms. Secondary endpoints as hospitalization days after completion of OI treatment, incidence of IRIS, virologic outcome at week 24 were also distributed equally in each treatment group. There were also no significant differences in self-reported bodily or psychological well-being using the SF36 body score and SF36 psychological score questionnaire between the two treatment groups.

Other trials with different study populations, mostly in resource poor countries and with different OI (like tuberculosis, cryptococcal meningitis) showed worse outcomes, if ART was initiated sooner, compared to deferred initiation [26, 28, 29].

The overall message regarding those OI (tuberculosis and cryptococcosis) was, not to defer ART at low CD4 cell counts, unless patients are suffering from neurologic affection, especially cryptococcal meningitis [34–37].

However, some further limitations deserve to be mentioned. Due to the small size of the two treatment groups, minor differences in primary and secondary endpoints of incidence rates of SAE might not have been detectable.

Another limitation is the fact, that nowadays a majority of patients is treated with an integrase inhibitor as the core agent in first-line ART. Data from the IDEAL trial probably cannot be applied for this treatment selection, because the incidence of treatment-associated immunological reconstitution might be higher with integrase inhibitors (INI). Therefore, it would be necessary to run another trial using INI as the core agent.

Furthermore, epidemiology, diagnostics and therapy in both PCP and TE are evolving, possibly leading to limitations in transferring the results to current populations [38, 39].

Despite a multicenter approach with much effort in 16 German HIV care experienced study centers only 61 of the planned 210 patients could get enrolled into the study. For the most part, in our opinion this was caused by the severity of the underlying condition (e.g. neurological alterations due cerebral toxoplasmosis, patients in critical conditions on intensive care units (e.g. patients couldn’t give informed consent in the short time period, which was mandatory for inclusion into the study). Additionally, difficulties occurred due to multiple potential drug interactions due to supportive drugs. E.g. ATV/r and proton pump inhibitors, often used in critical care patients, couldn’t be coadministered. These difficulties should be considered in the planning of potential future early treatment evaluation trials with novel integrase-based HIV regimens, that have fewer side effects.

## Conclusion

In summary, this study supports the hypothesis that immediate initiation of antiretroviral therapy with a ritonavir-boosted proteinase-inhibitor and a combination of two nucleoside reverse transcriptase inhibitors in treating the above mentioned OI is save and has no negative effects on incidence of disease progression or immune reconstitution inflammatory syndrome, nor on immunological and virologic outcome or on quality of life.

We have to emphasize the lack of both, other studies in this important field of HIV medicine and long-term observations. For long term analysis, monitoring the study population after several years is planned. Further investigations should investigate other OI and use other, modern ART-regimens.

## Data Availability

Full data is available.
